# LPS Disrupts Endometrial Receptivity by Inhibiting STAT1 Phosphorylation in Sheep

**DOI:** 10.3390/ijms252413673

**Published:** 2024-12-21

**Authors:** Xing Fan, Jinzi Wei, Yu Guo, Juan Ma, Meiyu Qi, He Huang, Peng Zheng, Wenjie Jiang, Yuchang Yao

**Affiliations:** 1 College of Animal Science and Technology, Northeast Agricultural University, Harbin 150038, China; fstar0213@163.com (X.F.); 13793309337@163.com (J.W.); jiegeng2023@163.com (Y.G.); mj212310@163.com (J.M.); huanghe@neau.edu.cn (H.H.); zhengpeng@neau.edu.cn (P.Z.); 2 Institute of Animal Husbandry, Heilongjiang Academy of Agricultural Sciences, Harbin 150086, China; joan7843@163.com

**Keywords:** LPS, endometrial receptivity, embryo implantation, STAT1, sheep

## Abstract

Uterine infections reduce ruminant reproductive efficiency. Reproductive dysfunction caused by infusion of Gram-negative bacteria is characterized by the failure of embryo implantation and reduced conception rates. Lipopolysaccharide (LPS), a major component of the outer membrane of Gram-negative bacteria, is highly abortogenic. In this study, the effects of LPS infusion on the endometrial receptivity of sheep were studied during three critical periods of embryo implantation. The results showed that LPS infusion on d12, d16, and d20 of pregnancy in vivo interfered with the expression of prostaglandins (PGs) and affected the expression of adhesion-related factors (*ITGB1/3/5, SPP1*), key implantation genes (*HOXA10*, *HOXA11* and *LIF*), and progestational elongation genes (*ISG15*, *RSAD2* and *CXCL10*) during embryo implantation. In addition, after LPS infusion on d12, d16, and d20, the phosphorylation level of STAT1 significantly decreased and the protein expression level of IRF9 significantly increased on d12, suggesting that LPS infusion in sheep impairs endometrial receptivity through the JAK2/STAT1 pathway. Sheep endometrial epithelial cells were treated with 17 β-estrogen, progesterone, and/or interferon-tau in vitro to mimic the receptivity of the endometrium during early pregnancy for validation. LPS and the p-STAT1 inhibitor fludarabine were both added to the model, which resulted in reduced p-STAT1 protein expression, significant inhibition of PGE2/PGF2α, and significant suppression of the expression of key embryo implantation genes. Collectively, these results indicate that LPS infusion in sheep on d12, d16, and d20 impairs endometrial receptivity through the JAK2/STAT1 pathway, which is responsible for LPS-associated pregnancy failure.

## 1. Introduction

Embryo implantation failure in early pregnancy is one of the main problems plaguing the benefits of livestock breeding and is a major limiting factor in assisted reproduction [1]. Successful embryo attachment mainly depends on high embryo quality, endometrial receptivity, and the interactions between the two. Abnormal endometrial receptivity is responsible for two-thirds of implantation failure cases [2]. Endometrial receptivity, which refers to the receptivity of maternal endometrium to blastocysts, is primarily regulated by ovarian-derived estradiol (E2), progesterone (P4), and conceptus-derived interferon-tau (IFN-τ) in ruminants.

Sheep have superficial/central implantation as opposed to rodents and primates, who have invasive implantation [3]. The process of implantation can be divided into three critical stages. In the first stage, on days 11–15 of pregnancy (d0 = zygote formation), after hatching from the zona pellucida, the ovoid conceptus of the sheep embryo, which is approximately 1 mm long on day 11, begins to elongate on day 12 and forms a filamentous conceptus 15 to 19 cm or more in length by day 15 [4,5]. Conceptus elongation is essential for maternal recognition of pregnancy as it maximizes IFN-τ production to prevent the production of luteolytic prostaglandin F2α (PGF2α) in the endometrial epithelium, thereby maintaining the corpus luteum and progesterone concentration [6]. Dynamic changes in the endometrial transcriptome occur between d7 and d13, which are primarily regulated by progesterone to modulate the components of uterine secretion to sustain endometrial functions and support the initiation of conceptus elongation [7]. In the second stage, on day 16, as the blastocyst develops into a filamentous conceptus, cellular contacts begin to be established between the embryonic trophoblast and the maternal endometrium [8]. There are significant changes in the transcriptomic profile of cell–matrix adhesion proteins, such as *ITGAV* and *ITGA6* [9], embryonic elongation genes, like *ISG15* and *OAS1* [10], and those involved in regulating immune response, with examples like *IL-12* and *IL-6* [11], as demonstrated by transcriptome studies on d15, d16, and d17 after insemination. In the third stage, on day 20 of pregnancy, the embryonic trophoblast adheres to the endometrial epithelium, completing implantation. Recent studies found that the endometrial transcriptome profile around the time of initiation of implantation (days 18–20) may be predictive of the ability of the conceptus to maintain pregnancy [12]. Key molecular events that occur in the process of maternal recognition of pregnancy are critically important for the successful establishment of pregnancy in ruminants.

The Janus kinase (JAK)/signal transducer and activator of transcription (STAT) signaling pathway plays an important role in the reproductive process [13]. During the “window of implantation” (WOI) in ruminants, IFN-τ activates the JAK2/STAT1/2 pathway, induces the expression of various interferon-stimulated genes (ISGs), mediates endometrial receptivity, and regulates pregnancy recognition [14]. The promotion of ISG expression by IFN-τ is mediated by ISG factor 3 (ISGF3), which consists of STAT1, STAT2, and interferon regulatory factor-9 (IRF-9) [15,16]. In cycling ewes, STAT1, STAT2, and IRF-9 mRNA and protein levels were low in the stroma (S) and glandular epithelium (GE) but high in the S and GE of the endometrium during early pregnancy, consistent with the temporal generation of IFN-τ [17]. P4 was found to increase IFN-τ expression in endometrial epithelial cells (EECs), which in turn increased the p-STAT1 and p-STAT3 levels [18]. The expression of STAT1 is significantly decreased during the formation of endometrial adenocarcinoma [19].

In animal husbandry, livestock are more susceptible to Gram-negative bacteria due to malnutrition, poor environmental conditions, decreased immunity during pregnancy, and reproductive trauma [20]. Lipopolysaccharide (LPS) is a major component of the cell wall of Gram-negative bacteria and is known as an endotoxin because it is released during normal metabolism and proliferation and after bacterial lysis [21,22]. The transcription levels of many genes involved in embryo–maternal interactions and implantation were altered significantly in bovine endometrial epithelial cells (BEECs) in response to LPS challenge in vitro [23]. Bacteria-induced endometrial inflammation alters histotroph composition and reduces conceptus growth and development on day 16 of pregnancy in cattle [24]. Most recently, LPS infusion has been shown to also affect the proliferation of the epithelium, GE, and S during implantation, causing reproductive disorders such as preterm labor, miscarriage, and reduced reproductive performance [25]. In addition, LPS has been reported to cause pyometra [26], endometrial damage [27], and implantation failure [28]. Despite an in-depth understanding of LPS-induced reproductive abnormalities in livestock, the role of LPS in endometrial receptivity is not well studied.

Therefore, we hypothesized that LPS infusion during early pregnancy damages the receptive endometrium of sheep and negatively affects embryo implantation. In the present study, we investigated the effects of LPS on embryo implantation failure by focusing on endometrial receptivity-related genes and PGs expression in sheep at different stages of implantation.

## 2. Results

### 2.1. Effects of LPS on the Endometrial Prostaglandin Expression Level in Sheep in the Three Periods

**Figure 1:**
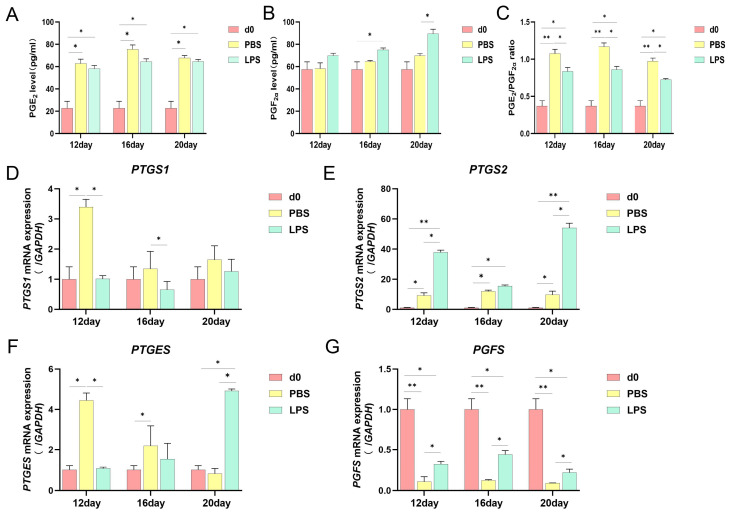
Effect of LPS on prostaglandin expression in sheep endometrium. (**A**) The secretion of PGE2 and PGF2α in endometrial tissue was measured on d12, d16, and d20 of pregnancy using an ELISA kit. (**B**) The secretion of PGE2 and PGF2α in endometrial tissue was measured on d12, d16, and d20 of pregnancy using an ELISA kit. (**C**) The ratio of PGE2 and PGF2α in endometrial tissue on d12, d16, and d20 of pregnancy. (**D**) The rate-limiting enzymes *PTGS1*, *PTGS2* (**E**), *PTGES* (**F**), and *PGFS* (**G**) of synthesized PGs in endometrial tissue on d12, d16, and d20 of pregnancy were measured by real-time quantitative PCR. All data are presented as the mean ± SEM, *n* ≥ 3; * *p* < 0.05; ** *p* < 0.01.

To examine the effect of LPS on the expression level of endometrial prostaglandin, PGE2 and PGF2α in endometrial tissue were detected using ELISA kits. Compared with d0, PGE2, and PGF2α showed higher levels in the d12, 16, and 20 PBS groups; after LPS treatment, the level of PGE2 did not significantly differ from the d12, 16, and 20 PBS groups, while the level of PGF2α was significantly elevated in the d16 and d20 LPS groups. After the infusion of LPS on d12, d16, and d20, the PGE2/PGF2α ratio significantly decreased (Figure 1A–C). Next, we studied the effect of LPS on prostaglandin-limiting enzymes in the endometrial tissue at three critical time points using real-time quantitative PCR. The results showed that from d12 to d20 in the PBS group, the mRNA expression level of *PTGS1* gradually decreased; after LPS treatment on d12, d16, and d20, compared with the PBS at these three time points, its levels were significantly reduced on d12 and d16 (Figure 1D). The mRNA expression level of *PTGS2* remained relatively stable and higher than d0 from d12 to d20 PBS; its level was significantly higher on d12 and d20 after LPS treatment (Figure 1E). From d12 to d20 in the PBS group, the mRNA expression level of *PTGES* gradually decreased; after LPS treatment on d12, d16, and d20, compared with the PBS at these three time points, its levels were significantly reduced on d12 and significantly increased at d20 (Figure 1F). The mRNA expression level of *PGFS* remained relatively stable and lower than d0 from d12 to d20 PBS; after LPS treatment, its levels significantly increased at all three time points (Figure 1G). The above results showed that LPS infusion interfered with prostaglandin secretion levels, disrupting the expression of prostaglandin-limiting enzymes during three periods of sheep implantation.

### 2.2. Effects of LPS on the Expression of Endometrial Implantation Genes in Sheep in the Three Periods

**Figure 2:**
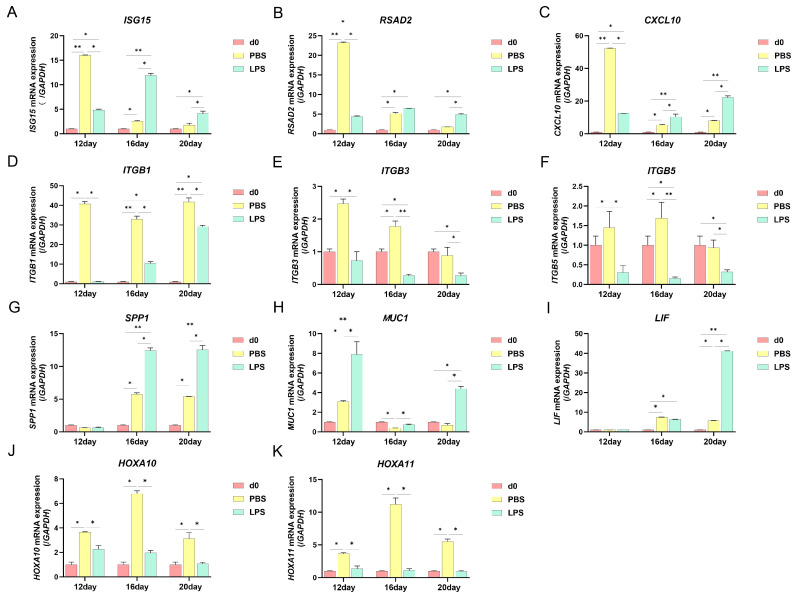
Effect of LPS on endometrial receptivity genes in sheep. (**A**) The pro-conceptus elongation gene *ISG15*, *RSAD2* (**B**), and *CXCL10* (**C**) on d12, d16, and d20 of pregnancy in endometrial tissue were measured by real-time quantitative PCR. (**D**) The adhesion molecules *ITGB1*, *ITGB3* (**E**), *ITGB5* (**F**), *SPP1* (**G**), and *MUC1* (**H**) on d12 of pregnancy in endometrial tissue were measured by real-time quantitative PCR. (**I**) The endometrial receptivity markers *HOXA10*, *HOXA11* (**J**), and *LIF* (**K**) on d12 of pregnancy in endometrial tissue were measured by real-time quantitative PCR. All data are presented as the mean ± SEM, *n* ≥ 3; * *p* < 0.05; ** *p* < 0.01.

To study the effect of LPS on endometrial receptivity during the three critical periods of implantation in sheep, the mRNA expression levels of key genes in endometrial tissues were measured using real-time quantitative PCR. The mRNA expression levels of the pro-pregnancy elongation genes *ISG15*, *RSAD2,* and *CXCL10* were decreased from d12 PBS to d20 PBS, respectively. The mRNA expression levels of *ISG15*, *RSAD2*, and *CXCL10* were significantly downregulated in the d12 LPS group compared with the d12 PBS group; the mRNA expression levels of *ISG15* and *CXCL10* were significantly upregulated in the d16, d20 LPS group compared with the d16, d20 PBS group; the mRNA expression levels of *RSAD2* in the d20 LPS group was significantly higher than that of the d20 PBS group (Figure 2A–C). Compared with the d0 PBS group, the mRNA expression levels of the adhesion-associated factors *ITGB1/3/5* were at a high level on d 12, d16, and d20; after LPS treatment, their expression levels significantly decreased (Figure 2D–F). In the d16 and d20 PBS groups as well as the d16 and d20 LPS groups, *SPP1* was significantly upregulated (Figure 2G). Compared with the d0 PBS group, the level of *MUC1* in the d16 PBS group was significantly downregulated. After LPS treatment, its levels were significantly elevated on d12, d16, and d20 (Figure 2H). LIF was significantly upregulated in the d20 LPS groups (Figure 2I). The mRNA expression levels of the attachment marker genes *HOXA10* and *HOXA11* were upregulated from d12 to d16 PBS and downregulated from d16 to d20 PBS. After treatment with LPS on d12, d16, and d20, the mRNA expression levels of *HOXA10* and *HOXA11* were significantly decreased (Figure 2J,K). These results indicate that the levels of genes associated with promoting conceptus elongation and cellular attachment were downregulated by LPS infusion on d12, d16, and d20, suggesting that LPS may damage the endometrial receptivity of sheep.

### 2.3. LPS Inhibits the JAK2/STAT1 Signaling Pathway

**Figure 3:**
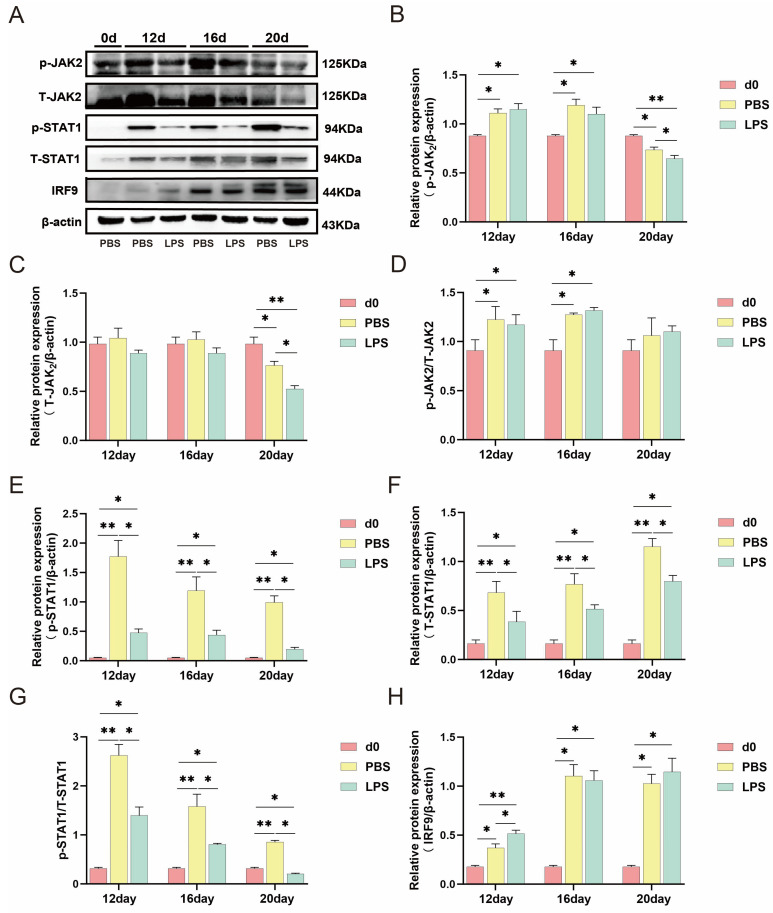
LPS affected JAK2/STAT1 pathways. (**A**) The protein level of p-JAK2, T-JAK2, p-STAT1, T-STAT1, and IRF9 on d12, d16, and d20 of pregnancy in sheep endometrial tissue. (**B**) p-JAK2/β-actin, T-JAK2/β-actin (**C**), and p-JAK2/T-JAK2 (**D**) ratio on d12, d16, and d20 of pregnancy in sheep endometrial tissue. (**E**) p-STAT1/β-actin, T-STAT1/β-actin (**F**), and p-STAT1/T-STAT1 (**G**) ratio on d12, d16, and d20 of pregnancy in sheep endometrial tissue. (**H**) The IRF9/β-actin ratio on d12, d16, and d20 of pregnancy in sheep endometrial tissue. All data are presented as the mean ± SEM, *n* ≥ 3; * *p* < 0.05; ** *p* < 0.01.

Previous studies suggested that the JAK2/STAT1 signaling pathway plays key roles in the regulation of IFN-τ during ruminant pregnancy. To clarify the mechanisms by which LPS affects endometrial receptivity, the expression of STAT1, p-STAT1, and IRF9 in the tissues was analyzed using Western blotting (Figure 3A). The results showed that compared with the d0 PBS group, the expression level of IRF9 and the p-STAT1/STAT1 ratio significantly increased in the d12 PBS, d16 PBS, and d20 PBS groups. After infusion with LPS at the three time points, the p-STAT1/STAT1 ratio decreased significantly while the IRF9 expression level increased in the d12 LPS group, but no significant difference was observed in the d16 LPS and d20 LPS groups (Figure 3E–H). JAK2, an upstream molecule of STAT1, was subsequently evaluated using Western blotting. However, LPS did not significantly alter the p-JAK2/T-JAK2 ratio on d12, d16, and d20 (Figure 3B–D), indicating that LPS infusion inhibited the JAK2/STAT1 signaling pathway.

### 2.4. Establishment of a Receptive Cell Model

**Figure 4:**
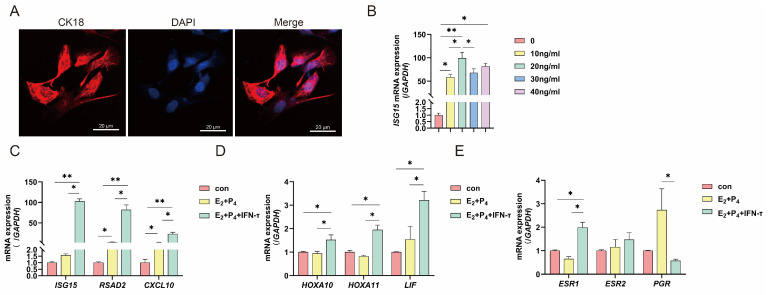
Establishment of a receptive sheep endometrial epithelial cell model for sheep. (**A**) Confocal microscopy was used to observe the morphology of sEECs. Red: Cy3-labeled cytokeratin 18 protein; blue, DAPI-labeled nuclei; scale bar: 20 µm. (**B**) Expression of ISG15 was measured under different concentrations in sEECs. (**C**–**E**) The endometrial receptivity-related genes *ISG15*, *RSAD2*, *CXCL10*, *HOXA10*, *HOXA11*, *LIF*, *ESR1*, *ESR2*, and *PGR* in sEECs were measured by real-time quantitative PCR. GAPDH (sheep) was used as the reference gene in all samples. sEECs: sheep endometrial epithelial cells. All data are presented as the mean ± SEM, *n* ≥ 3; * *p* < 0.05; ** *p* < 0.01.

The mechanisms by which LPS affects endometrial receptivity in sheep were also explored in vitro. We treated sEECs with hormones in vitro to mimic the receptive state of the uterus and detected the mRNA expression levels of the proembryonic elongation factors *ISG15*, *CXCL10*, and *RSAD2*, the key attachment genes *HOXA10*, *HOXA11*, and *LIF*, and the hormone receptors *ESR1*, *ESR2*, and *PGR*. sEEC integrity was determined using a keratin 18-specific antibody (CK18) (Figure 4A). To determine the appropriate IFN-τ concentration, sEECs were treated with 0, 10, 20, 40, and 80 ng/mL for 12 h. Treatment with IFN-τ at a concentration of 20 ng/mL significantly increased the *ISG15* transcription level. No significant difference was found in the *ISG15* transcription level between high concentration (40 and 80 ng/mL) and 20 ng/mL IFN-τ treatments (Figure 4B). Therefore, 20 ng/mL IFN-τ was selected for subsequent experiments. The results showed no significant differences, except for a significant increase in the mRNA levels of *RSAD2*, *CXCL10,* and *PGR* upon treatment with E2 and P4 alone. After IFN-τ treatment, *ISG15*, *CXCL10*, *RSAD2*, *HOXA10*, *HOXA11*, *LIF,* and *ESR1* mRNA levels significantly increased, *ESR2* mRNA level did not change significantly, and PGR mRNA level significantly decreased (Figure 4C–E). The above results indicate that the combined treatment of sheep endometrial epithelial cells with E2, P4, and IFN-τ could mimic the receptive endometrium.

### 2.5. LPS Damaged Sheep Endometrium by Inhibiting the JAK2/STAT1 Pathway

**Figure 5:**
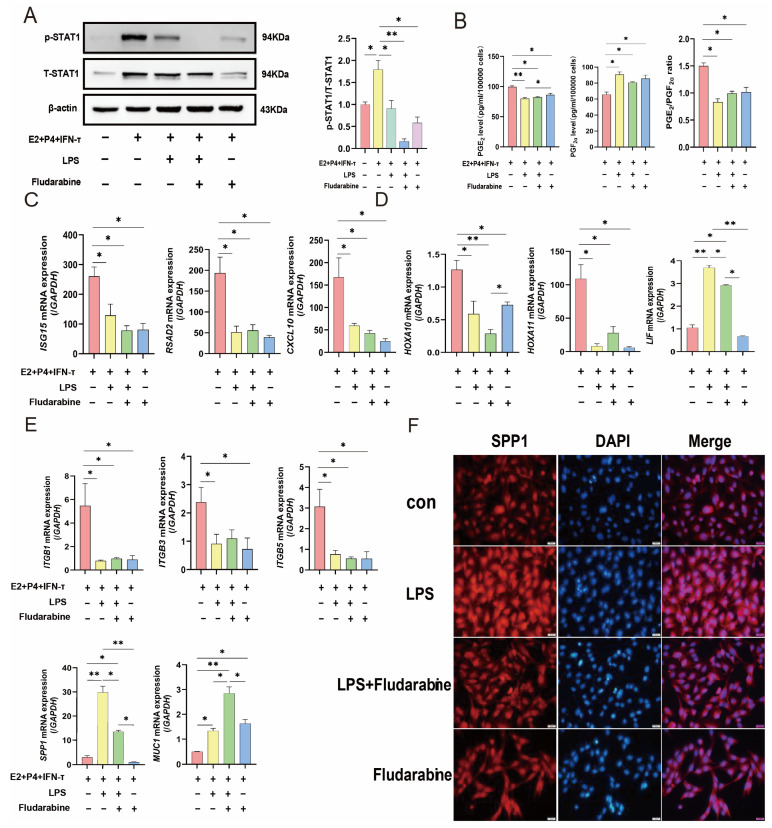
Effect of LPS or fludarabine treatment on the expression of endometrial receptivity-related genes under hormone treatment. (**A**) The protein level of p-STAT1 and T-STAT1 in sEECs. (**B**) The secretion of PGE2 and PGF2α in sEECs. (**C**–**E**) The pro-conceptus elongation genes *ISG15*, *RSAD2*, *CXCL10*, adhesion molecules *ITGB1/3/5*, *MUC1*, *SPP1*, and receptivity markers *HOXA10*, *HOXA11*, *LIF* mRNA expression levels in sEECs. GAPDH (sheep) was used as the reference gene in all samples. (**F**) Confocal microscope images of SPP1 expression in four treatment groups. Red: Cy3-labeled SPP1 protein; blue, DAPI-labeled nuclei; scale bar: 20 µm. All data are presented as the mean ± SEM, *n* ≥ 3; * *p* < 0.05; ** *p* < 0.01.

We used the STAT1 inhibitor fludarabine to inhibit the JAK2/STAT1 pathway to investigate whether LPS affects the endometrium of sheep. Western blotting results showed that the expression of p-STAT1 was significantly inhibited by the addition of fludarabine compared with the model group, and the expression of p-STAT1 was significantly reduced after treatment with 5 µg/mL LPS, suggesting that LPS can significantly inhibit STAT1 phosphorylation in the cell experiments. Prostaglandin secretion was detected using the kit, and the results showed that compared with the model group, the secretion level of PGE2 significantly decreased, while that of PGF2α significantly increased, and the PGE2/PGF2α ratio significantly decreased in the LPS treatment group. In the fludarabine group, the secretion level of PGE2 significantly decreased, while that of PGF2α significantly increased, and the PGE2/PGF2α ratio significantly decreased. The fludarabine and LPS cotreatment groups showed similar changes (Figure 5B). RT-PCR was used to detect changes in the tolerance-related genes. The results showed that compared with the model group, the addition of LPS significantly decreased the expression of tolerance-related genes, except for *LIF*, *SPP1,* and *MUC1*. The same results were observed after the addition of fludarabine alone or after co-treatment with LPS and Flu. *MUC1* expression significantly increased after the addition of LPS or fludarabine and was even more significantly increased after co-treatment with LPS and fludarabine (Figure 5C–E). After the addition of LPS, *LIF*, and *SPP1* expression significantly increased. In contrast to LPS, the expression of *LIF* and *SPP1* mRNA decreased, but not significantly, after the addition of fludarabine. *SPP1* and *LIF* levels also increased significantly after co-treatment (Figure 5D–F). Taken together, these data indicate that LPS infusion disrupts the endometrial receptivity of sheep by inhibiting the JAK2/STAT1 signaling pathway.

## 3. Discussion

In this study, we examined the detrimental effects of LPS exposure on endometrial receptivity in sheep. Our results indicated that the adverse effect of LPS on the expression of endometrial receptivity-related genes and prostaglandin levels was extensive, potentially leading to failure of embryo implantation in sheep.

PGF2α is a factor that induces luteolysis, whereas PGE2, which has the opposite function to PGF2α, inhibits luteolysis [29]. Therefore, an appropriate PGE2/PGF2α ratio is important for endometrial receptivity [30]. LPS treatment caused preterm labor in ICR mice, with an upregulation of uterine COX-2 and a corresponding increase in maternal serum concentrations of PGF2α [31]. Administration of 0.5 mg/kg LPS to circulating dairy cows can cause temporary inhibition of luteal function and morphology and increase PGF2α metabolite concentration [32]. In the present study, the levels of PGE2 significantly increased on d12, d16, and d20 of normal pregnancy whereas those of PGF2α did not change significantly. The PGE2/PGF2α ratio was significantly higher at all the three time points compared with d0 PBS, suggesting a tendency for a significant increase in the ratio during the early stages of pregnancy in sheep. After LPS infusion on d12, d16, and d20 of pregnancy, the PGE2/PGF2α ratio decreased significantly, suggesting that LPS infusion can lead to dysregulation of prostaglandin secretion in the endometrium. Additionally, injection of meloxicam, an inhibitor of PTGS2, into the uterus of sheep during early pregnancy hindered the elongation of the pregnant body [33,34]. Stimulation of the uterine arteries with LPS results in a significant increase in PTGS2 protein levels, which suggests that uterine arteries utilize PTGS2 to respond to pathological stimuli, such as LPS, resulting in prostacyclin release [35]. In this study, we found that *PTGS1* expression significantly increased, mainly on d12 and d20, and *PTGS2* expression significantly increased on d16 and d20 compared with d0 PBS. After LPS infusion at the three time points, *PTGS1* expression significantly decreased on d12 and d16 and *PTGS2* expression significantly increased on d12 and d20. *PTGES* expression was significantly higher on d12 and d16 of normal pregnancy compared with d0 PBS. After LPS infusion, *PTGES* expression was significantly lower in the d12 LPS group, decreased, although not significantly, in the d16 LPS group, and significantly increased in the d20 LPS group. The different results on PTGES after LPS infusion at the three periods may be explained by different critical events in each period.

ISGs may also be involved in the regulation of endometrial receptivity, conceptus elongation, and implantation [36,37]. High expression of *ISG15* has been found to promote conceptus elongation [38] and immune cell proliferation and increase IFN-τ secretion [39]. *ISG15* was highly expressed in luminal epithelial (LE) of sheep uterus on day 11 or 12 of pregnancy [37]. High expression of *CXCL10* induces the recruitment of a large number of immune cells to the uterus and promotes blastocyst adhesion to the uterine endometrium [40]. In pregnant ewes, the mRNA levels of *RSAD2* are significantly elevated in the endometrium, mesenchyme, and immune cells [41]. Additionally, endometrial *RSAD2* was significantly elevated between days 12 and 16, but not of the estrous cycle [41]. In the present study, the mRNA expression levels of *ISG15* and *RSAD2* significantly increased in the d12 PBS and d16 PBS groups compared with the d0 PBS groups, while that of *CXCL10* was significantly higher at all three time points. After LPS infusion on d12, the expression levels of *ISG15*, *RSAD2,* and *CXCL10* decreased, indicating that the infusion of LPS on d12 may have inhibited the elimination of the conceptus. However, the expression levels of these three genes increased after LPS infusion on d16 and d20. Studies have shown that LPS promotes the release of free *ISG15* from monocytes and lymphocytes [42]. *CXCL10* mRNA levels were upregulated in uterine cervical fibroblasts after LPS stimulation [43]. Therefore, these opposing results of LPS infusion between d12, d16, and d20 may suggest that *ISG15*, *RSAD2*, and *CXCL10* also act as anti-inflammatory factors in vivo. Numerous adhesion molecules and extracellular matrix components, such as integrins, *SPP1*, and *MUC1*, play important regulatory roles during adhesion [44,45]. *ITGB1* is abundant in endometrial tissues at the maternal–fetal interface [1,6]. *ITGB3* is present in GE, LE, and S, and its relative mRNA expression levels gradually increase on d16 of pregnancy and begin to decrease on d17 [8]. We found that *ITGB1/3/5* levels were significantly higher in the d12 and d16 groups than in the d0 PBS group during pregnancy, whereas only the *ITGB1* level was significantly elevated in the d20 PBS group. *ITGB1/3/5* expression significantly decreased after LPS infusion on d12 and d16, and *ITGB3/5* expression significantly decreased in the d20 LPS group. SPP1 binds to integrin receptors expressed in endometrial LE and mediates the adhesion between the LE and trophoblasts [46,47]. *SPP1* mRNA was detected in some uterine glands on d13 of pregnancy and was expressed in all glands on d19 [6,48]. We found that *SPP1* expression significantly increased on d16 and d12, but *SPP1* showed an upward trend after LPS treatment on d16 and d20. In vivo and in vitro, *SPP1* was upregulated with the increase in NF-kB activity, and LPS treatment of bovine mammary epithelial cells increased *SPP1* expression [49]. Our findings are consistent with those of previous studies. In rodents and pigs [50], *MUC1* expression in the uterine LE decreased before implantation and increased during the receptive period in rabbits and humans [51,52], but it may be partially reduced at the attachment site of pregnancy. *MUC1* in LE of sheep is significantly reduced on day 9 of pregnancy and is almost undetectable on day 17 [53]. Our results showed that the *MUC1* expression significantly decreased in the d16 PBS group. After LPS infusion, *MUC1* expression significantly increased at all three time points. These results suggest that LPS reduces the ability of the endometrium to adhere to the conceptus by d16 of pregnancy. Recent studies have shown that *HOXA10*, *HOXA11,* and *LIF* are used to evaluate endometrial receptivity [54,55]. *HOXA10* can affect the expression of uterine receptivity-related media, such as integrin avB3 and estrogen receptors [56]. *HOXA11* is involved in embryo implantation, with its highest expression levels observed during WOI [57]. We found that *HOXA10* and *HOXA11* levels were significantly elevated on d12, d16, and d20 in a normal pregnancy but were significantly reduced at all three time points after LPS infusion. *LIF* regulates endometrial receptivity through integrins *ITGB3* and *ITGB5* [58]. Its reduced expression leads to pregnancy failure [59,60]. The administration of LPS to mice on day 7 of gestation resulted in 100% embryonic resorption accompanied by an increase in *LIF*, which appears to be a mediator of progesterone action under inflammatory conditions [61]. On d16 and d20 of normal pregnancy, the *LIF* level was significantly elevated compared with the d0 PBS group, whereas LPS infusion on d20 resulted in a significant increase in *LIF*. These results indicate that LPS infusion on d12, d16, and d20 causes some degree of damage to the endometrial receptivity in sheep.

ISGF3 drives type I IFN-stimulated transcriptional activation [13], which involves SH2-phosphotyrosine-mediated STAT1 and STAT2 associated with IRF9 in ruminants [62]. In the uterus of bacteria-infected ruminants, peptidoglycans from antigenic cells can lead to early pregnancy embryo loss by inhibiting STAT1 expression [63]. To determine the molecular mechanism by which LPS impairs endometrial receptivity in sheep, we evaluated the protein levels of the JAK2/STAT1 signaling pathway. The results showed that the p-STAT1/T-STAT1 ratio and IRF9 protein expression significantly increased on d12, d16, and d20 of normal pregnancy compared with d0 PBS. After LPS infusion during these three periods, the p-STAT1/T-STAT1 ratio significantly decreased and IRF9 protein expression significantly increased. Furthermore, we detected JAK2, a protein upstream of the STAT1 pathway. The results showed that p-JAK2/T-JAK2 significantly increased on d12 and d16 in normal pregnancy whereas p-JAK2/T-JAK2 did not change significantly after LPS infusion. Additionally, the phosphorylation of STAT1 was significantly inhibited by LPS infusion on d12, d16, and d20.

**Figure 6:**
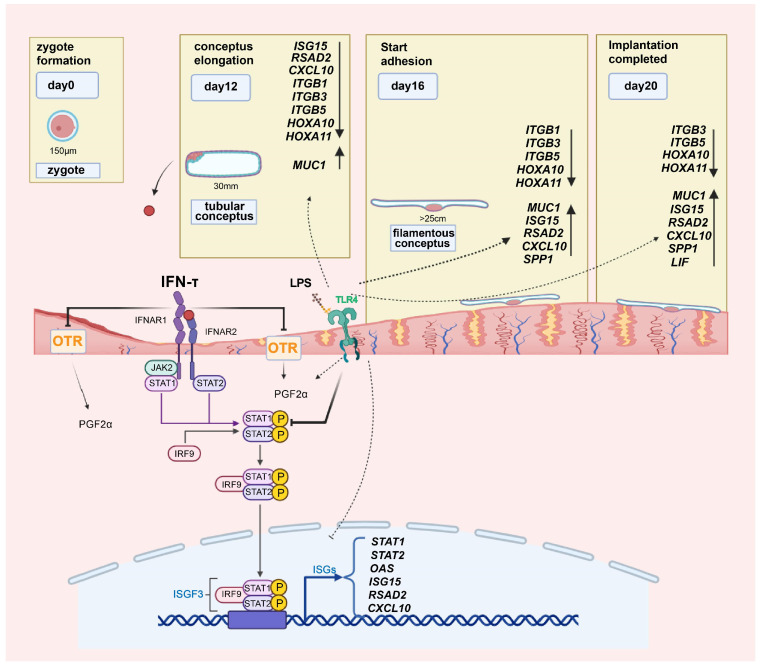
Schematic characterization of the cellular mechanism of LPS infusion effects on endometrial receptivity in sheep during early pregnancy. LPS blocked the effect of IFN-τ in the three stages of sheep embryo implantation and impaired the endometrial receptivity, which is characterized by interfering with the secretion of prostaglandins, hindering the elongation of the conceptus, and reducing the adhesion of the embryo by inhibiting the phosphorylation of STAT1.

Furthermore, sEECs were treated with E2, P4, and IFN-τ in vitro to construct a receptive cell model for validation [30]. The results showed that after hormone combination treatment, the expression of *ISG15*, *RSAD2*, *CXCL10*, *HOXA10*, *HOXA11*, *LIF,* and *ESR1* significantly increased, while that of *PGR* significantly decreased, which was in accordance with the trend in normal pregnancy. The above results indicate that the combination treatment of sheep endometrial epithelial cells with E2, P4, and IFN-τ can simulate the receptive endometrium to a certain extent. We further determined whether LPS affected endometrial receptivity by inhibiting the JAK1/STAT1 pathway. The p-STAT1 inhibitor fludarabine was used to treat receptive sEECs. Compared with the control, PGE2 expression significantly decreased, PGF2α expression significantly increased, and the PGE2/PGF2α ratio significantly decreased in the LPS, fludarabine. and co-treatment groups. Both LPS and fludarabine treatments significantly reduced the mRNA expression of *ISG15*, *RSAD2*, *CXCL10*, *HOXA10*, *HOXA11*, and *ITGB1/3/5*. Co-treatment with LPS and fludarabine further reduced the expression of *HOXA10*. *MUC1* expression significantly increased after the addition of LPS or fludarabine and was even more significantly elevated after co-treatment with the two. *LIF* and *SPP1* levels did not change significantly after the addition of fludarabine but were significantly elevated after the addition of LPS. These results indicate that LPS disrupts endometrial receptivity by inhibiting STAT1 phosphorylation, which affects embryo implantation in sheep (Figure 6).

Although this study combines in vivo (LPS infusion in sheep in vivo) and in vitro (treatment of sheep endometrial epithelial cells) experiments, the in vivo environment is complex and in vitro simulation is difficult to fully replicate the real situation in vivo, which may differ from the actual situation. But, this study provides an important scientific basis for improving reproductive efficiency in livestock by revealing how LPS affects endometrial receptivity in domestic animals and understanding the cellular and molecular signals that regulate uterine receptivity and implantation.

## 4. Materials and Methods

### 4.1. Animal Tissue Collection

Healthy and undelivered 12-month-old ewes were selected as research subjects. Thirty-five ewes with normal estrous cycles and body weight of 45–55 kg were selected (each group of five ewes represented a time point). Controlled internal drug release (CIDR) was used to induce estrus synchronization in ewes buried for 14 d. At the same time, 0.1 mg PG and 330 IU pregnant mare serum gonadotropin were intramuscularly injected. From the 36th hour after the withdrawal of the CIDR intravaginal device, the rams were tested every 8 h. Artificially collected semen was diluted with sterile dilution fluid (prepared with endotoxin-free water) to a concentration of 2.5 × 10^8^/mL. The specific time point of animal tissue collection is shown in Supplementary Figure S1. Twelve hours after the ewe entered estrus, deep intrauterine artificial insemination into the ovulatory uterine horn was performed using a laparoscope, with 0.1 mL of diluted semen being inputted into the ovulatory uterine horn. Four sampling time points were selected according to the different stages of sheep embryo implantation: zygote formation (d0); conceptus elongation (d12); establishment of structural links between the embryo and the uterine epithelium (d16); and completion of implantation (d20). Of these four time points, the three key nodes d12, d16, and d20 were selected, and a 1 mg/mL LPS (Escherichia coli O111:B4) concentrate was first configured, followed by dilution of 80 µL of LPS concentrate with 1.52 mL of phosphate-buffered saline (PBS). Twenty-four hours before the uterine tissue sampling, LPS (treatment group) or PBS (control group) (0.8 mL) was perfused into the uterus via laparoscopy combined with a uterine horn insemination gun. The remaining five individuals at each node received the same amount of PBS or LPS perfusion. After anesthetizing the ewes, the entire uterus was removed and longitudinally sectioned under sterile conditions. After longitudinal uterine incision, the success of the pregnancy (presence of an embryo on d12, d16, and d20) was first confirmed. Then, the uterus was flushed with PBS and maternal endometrial tissue samples were taken at the maternal–fetal interface, maintaining a thickness of 1 mm. The tissues were immediately transferred to a 2 mL DNase- and RNase-free tube filled with RNAlater (Qiagen, Valencia, CA, USA) and stored at −80 °C.

### 4.2. Cell Culture and Drug Treatment

The sheep endometrial epithelial cells (sEECs) were kindly provided by Hongbing Han from the laboratory of the College of Animal Science and Technology, China Agricultural University. Culture conditions used in this study are shown in Supplementary Figure S2 [30]. The sEECs were seeded in a dish containing high-glucose Dulbecco’s modified eagle medium (DMEM) supplemented with 10% fetal bovine serum (Pricella Biotechnology Co., Ltd., Wuhan, China.) and incubated at 37 °C in a humidified 5% CO_2_ incubator. The culture medium was replaced every day. The sEECs were seeded in six-well plates, and upon reaching a cell density of 70–80% according to Yang’s description [30], the EECs were cultured in fresh high-glucose DMEM plus 0.1% bovine serum albumin (BSA) for 12 h. Then, P4 (10^−7^ M, Sigma-Aldrich, St. Louis, MO, USA) and E2 (10^−9^ M, Sigma-Aldrich, St. Louis, MO, USA) were added to the medium. After hormone treatment for 12 h, the EECs were treated with 20 ng/mL recombinant ovine interferon-tau (C600063; Sangon Biotech, Shanghai, China) for 12 h. Then, 5 µg/mL LPS (L4391; Sigma-Aldrich) was added to the medium. In the presence of fludarabine (MedChemExpress, Monmouth Junction, NJ, USA), 10 μM fludara was added to the EECs before adding LPS for 2 h.

### 4.3. PG Measurement

Tissue samples were lysed with PBS and protease inhibitor cocktail (1:100; MedChemExpress) via grinding. After centrifugation of the samples, the supernatant was collected and stored at −80 °C. After 12 h of LPS stimulation of cells, 1.0 mL of cell supernatant was collected and stored at −80 °C until analysis. The concentrations of PGF2α and PGE2 were measured using a sheep PGF2α ELISA kit (JYM0123sh; JYM Supplement Science, Wuhan, China) or goat PGE2 ELISA kit (JYM0120Go; JYM Supplement Science, Wuhan, China) according to the manufacturer’s instructions.

### 4.4. RNA Extraction and Real-Time Quantitative Polymerase Chain Reaction

Total RNA was isolated from the endometrial tissue or endometrial epithelial cells using the Cell Total RNA Isolation Kit (Foregene, Chengdu, China) according to the manufacturer’s instructions. The RNA concentration and purity were assessed using a NanoDrop spectrophotometer (Thermo Fisher Scientific, Waltham, MA, USA). RNA (1000 ng) was reverse transcribed, and cDNA was prepared using ABclonal kits (RK20409, ABclonal Technology Co., Ltd., Wuhan, China). Real-time qPCR was performed on a LightCycler 480 Real-Time Detection System (Roche, Basel, Switzerland). Each reaction mixture consisted of 5 µL of ROX (4913850001; Roche, Basel, Switzerland), 0.3 µL each of forward and reverse primers (0.3 μM), 1.0 μL of cDNA (1000 ng), and distilled water up to a final volume of 10 µL. Primer sequences for the target genes are listed in Supplemental Table S1. Amplification cycle was programmed as follows: 95 °C for 30 s, followed by 40 cycles of 95 °C for 10 s, 60 °C for 20 s, and 72 °C for 10 s. GAPDH (sheep) was used as the reference gene, and the relative expression of each gene was calculated using the 2^−ΔΔCT^ method.

### 4.5. Western Blot Analysis

Cells and tissue samples were lysed using radioimmunoprecipitation assay buffer (Beyotime Biotechnology, Shanghai, China) containing 1:100 (*v*/*v*) phenylmethylsulfonyl fluoride (Roche) and 1:50 (*v*/*v*) protease and phosphatase inhibitor cocktail (Beyotime Biotechnology, Shanghai, China) on ice for 15 min to obtain protein samples. The protein concentration of each sample was measured using a BCA Protein Assay Kit (Beyotime Biotechnology, Shanghai, China). Proteins mixed with the loading buffer were incubated at 100 °C for 10 min and separated using 10–12% sodium dodecyl sulfate-polyacrylamide gel electrophoresis. Separated proteins were transferred onto polyvinylidene fluoride membranes (Millipore, Billerica, MA, USA). The membranes were blocked with 5% BSA in Tris-buffered saline with Tween 20 at 37 °C for 60 min and incubated overnight at 4 °C with primary antibodies against STAT1 (1:2000; Cell Signaling Technology, Danvers, MA, USA), p-STAT1 (1:2000; Cell Signaling Technology, Danvers, MA, USA), JAK2 (1:2000; Cell Signaling Technology, Danvers, MA, USA), p-JAK2 (1:1000; Cell Signaling Technology, Danvers, MA, USA), and IRF9 (1:2000; Proteintech, Rosemont, IL, USA), with β-actin (1:3000; AF7018; Affinity Biosciences, Cincinnati, OH, USA) as an internal reference. The membranes were washed and incubated for 1 h with a horseradish peroxidase-conjugated secondary antibody (1:2000; A0208; Beyotime Biotechnology, Shanghai, China) at room temperature. Chemiluminescent detection reagents (Cat No. 34577; Thermo Fisher Scientific, Cleveland, OH, USA) were used to visualize the immunoblots, and the relative intensities were analyzed using ImageJ software (version 1.45; National Institutes of Health, Bethesda, MD, USA).

### 4.6. Immunofluorescence Staining

The cells were washed thrice with pre-cooled PBS and fixed with 4% paraformaldehyde for 15 min. The cells were permeabilized for 30 min with 0.5% Triton X-100. Subsequently, the permeabilized cells were blocked with 2% BSA for 1 h and incubated with primary antibodies against SPP1 (1:200; WL02378; Wanleibio Co., Ltd., Shenyang, China) and cytokeratin 18 (1:200; AF0191; Affinity Biosciences, Cincinnati, OH, USA) at 4 °C overnight. After washing, the cells were incubated with fluorescently labeled secondary antibodies (1:200; A0516; Beyotime Biotechnology, Shanghai, China) for 80 min at room temperature. The nuclei were stained using DAPI (Beyotime Biotechnology, Shanghai, China), and the images were obtained under a confocal microscope.

### 4.7. Statistical Analysis

Cell culture experiments were repeated at least thrice. Statistical analyses were performed using SPSS 26.0 (IBM Corporation, Armonk, NY, USA). All data were subjected to a one-way analysis of variance, followed by the Waller–Duncan test and Fisher’s least significant difference test. Quantitative data are presented as the mean ± standard error of the mean, and statistical significance was set at *p* < 0.05.

## 5. Conclusions

In summary, LPS infusion during early pregnancy interferes with the expression of PGs in the endometrium and reduces the expression of receptivity-related genes by inhibiting STAT1 phosphorylation, ultimately affecting embryo implantation in sheep.

## Data Availability

Data is contained within the article and Supplementary Materials.

## Supplementary Materials

The following supporting information can be downloaded at: https://www.mdpi.com/article/10.3390/ijms252413673/s1.

## Abbreviations

LPS: Lipopolysaccharide; PGs: Prostaglandins; E2: Ovarian-derived estradiol; P4: Progesterone; IFN-τ: Interferon-tau; JAK: Janus kinase; STAT: Signal transducer and activator of transcription; WOI: Window of implantation; ISGs: Interferon-stimulated genes; ISGF3: ISG factor 3; IRF-9: Interferon regulatory factor-9; S: Stroma; GE: Glandular epithelium; EECs: Endometrial epithelial cells; BEECs: Bovine endometrial epithelial cells.
