# Supplementary material for: LPS Disrupts Endometrial Receptivity by Inhibiting STAT1 Phosphorylation in Sheep
Source: Int J Mol Sci. 2024 Dec 21;25(24):13673. doi: 10.3390/ijms252413673 (PMC11678167; doi:10.3390/ijms252413673)
Supplement: Supplementary file 1 [file ijms-25-13673-s001.zip › ijms-3331954-supplementary.pdf]

Supplemental Table S1. List of primers used in quantitative RT-PCR.

Gene symbol	GenBank accession no.	Forward primer sequence (5′ → 3′)	length	Tm	Reverse primer sequence (5′ → 3′)	length	Tm	product length
ISG15	NM_001009735	GGCCATGGGCGGGGA	15	61.26	TTCTGGGCGATGAACTGCTT	20	59.96	108
RSAD2	XM_004005669	CACCAGCGTCAATTACCACTC	21	59.27	TTCAGCATCAGCAGACCTCTC	21	59.79	123
CXCL10	NM_001009191	TTGTTCGTGGACTTCAGTTCTC	22	58.55	AAGGCTGGGACTTAGCACATTA	22	59.42	185
ITGB1	NM_001113770	TAGAGACTCCAGAGTGCCCC	20	60.03	CCGTGTCCCATTTGGCATTC	20	59.83	180
ITGB3	XM_060395753	GGTGGTCTTGCTATCCGTGA	20	59.46	GTTGTTGGCAGTGTCCCATG	20	59.69	151
ITGB5	XM_027956587	GGGTCCGATGTCATCCAGC	19	60.23	GAACGTAGTCTTGTCACCGGG	21	60.67	72
MUC1	XM_027976040	TCTCATTGCCCTGGTTGTGT	20	59.52	TAGGGGCTCCGTTTGGTACT	20	60.25	156
SPP1	NM_001009224	ACTGCATCAGCATCACAGGG	20	60.39	TCGGTTTAACTGGAAGGGCG	20	60.32	131
HOXA10	XM_004007939	GTACCTTACTCGAGAGCGGC	20	59.97	TTGCCTGGAGCTTCATCAGG	20	60.03	172

HOXA11	XM_015095335	CTACGTCTCGGGTCCAGATT	20	58.61	CATTTAGTGGCGGGCTCA	18	57.36	159
LIF	XM_012098046	TATCGCATCATCGCGTACCT	20	59.12	ACATGGCTCACGTGGTACTT	20	59.31	176
PTGS1	NM_001009476	CGCTGGCTTTGGGATTTTGT	20	59.68	AGGTTGGAACGCACTGTGA	19	59.48	80
PTGS2	NM_001009432	TCCATGCCAGAATCGAGGTG	20	59.82	GTGTCGGGAGTGGGTTTCAG	20	60.60	147
PTGES	XM_027966307	GCTGCGGAAGAAGGCTTTTG	20	60.39	CGGTCCGAGGAAGGAGTAGA	20	60.11	169
PGFS	XM_012188608	ATGGATCCCAAAAGCCAGAGG	21	60.06	AAGTGCACCAAAGCTCCTCA	20	59.82	97
ESR1	XM_042253635	GGCTACGCAAGTGCTATGA	19	57.64	TGTGCTTCAACATTCTCCCT	20	57.04	81
ESR2	NM_001009737	ATCCATTGGCAGTCGTCA	18	56.21	CTCCCACTAGCTTTCCTTTT	20	54.94	122
PGR	XM_015100878	GATTCAGAGCCAGCCAGAG	19	57.62	GCAATCGTTTCTTCCAGCAC	20	58.03	185
GAPDH	NM_001190390	CAAGTTCCACGGCACAGTCA	20	60.81	TGGTTCACGCCCATCACAA	19	59.85	249

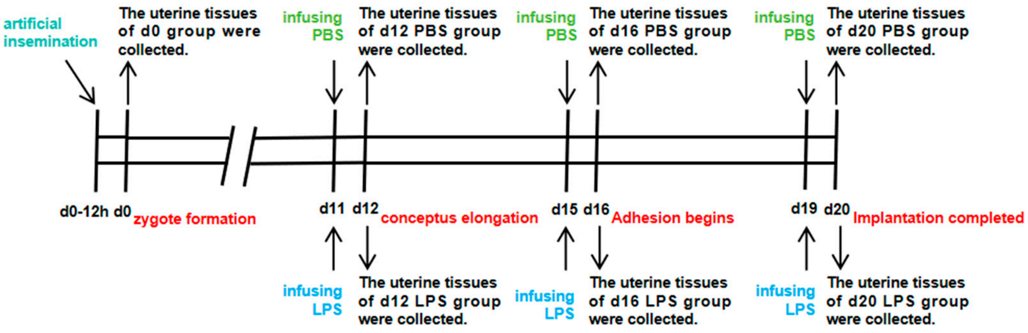

Supplemental Figure S1: Schedule of the Animal Tissue Collection. Twelve hours after artificial insemination was recorded as day 0, and d0 uterine tissue was collected as d0 group. At d11, The ewes were received the same amount of PBS or LPS perfusion, and the uterine tissues were taken on d12 as d12 PBS group and d12 LPS group, respectively. At d15, The ewes were received the same amount of PBS or LPS perfusion, and the uterine tissues were taken on d16 as d16 PBS group and d16 LPS group, respectively. At d19, The ewes were received the same amount of PBS or LPS perfusion, and the uterine tissues were taken on d20 as d20 PBS group and d20 LPS group, respectively.

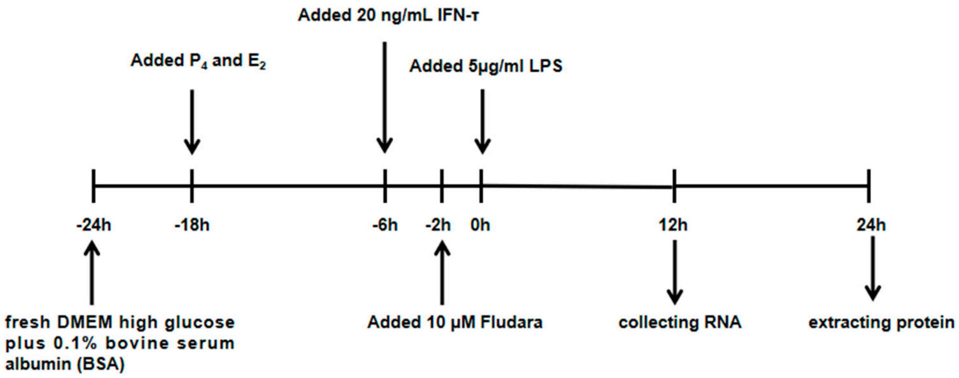

Supplemental Figure S2: Schedule of the sEECs treatment. From -24 to -18 h, sEECs was grown in fresh DMEM high glucose plus 0.1 % BSA; -18 to -6 h is the stage in which maternally derived P4 and E2 were added to mimic the proliferative phase; -6-0h is the stage in which conceptus-derived IFN-τ was added to mimic maternal recognition of pregnancy. 0 h is the time of LPS addition. 10 μM Fludara was added to the sEECs before adding LPS for 2 h. RNA and protein were extracted at 12h and 24h respectively. “-” represents pretreatment before the time point (0h).
